# Development and validation of an RNA binding protein-associated prognostic model for hepatocellular carcinoma

**DOI:** 10.1186/s12885-020-07625-3

**Published:** 2020-11-23

**Authors:** Min Wang, Shan Huang, Zefeng Chen, Zhiwei Han, Kezhi Li, Chuang Chen, Guobin Wu, Yinnong Zhao

**Affiliations:** 1 Department of Hepatobiliary Surgery, Guangxi Medical University Cancer Hospital, Nanning, China; 2 Department of Chinese Medicine, Guangxi Medical University Cancer Hospital, Nanning, China; 3 Department of Experimental Research, Guangxi Medical University Cancer Hospital, Nanning, China

**Keywords:** RNA binding protein, Hepatocellular carcinoma, Prognosis, Comprehensive bioinformatics analysis

## Abstract

**Background:**

Hepatocellular carcinoma (HCC) is among the deadliest forms of cancer. While RNA-binding proteins (RBPs) have been shown to be key regulators of oncogenesis and tumor progression, their dysregulation in the context of HCC remains to be fully characterized.

**Methods:**

Data from the Cancer Genome Atlas - liver HCC (TCGA-LIHC) database were downloaded and analyzed in order to identify RBPs that were differentially expressed in HCC tumors relative to healthy normal tissues. Functional enrichment analyses of these RBPs were then conducted using the GO and KEGG databases to understand their mechanistic roles. Central hub RBPs associated with HCC patient prognosis were then detected through Cox regression analyses, and were incorporated into a prognostic model. The prognostic value of this model was then assessed through the use of Kaplan-Meier curves, time-related ROC analyses, univariate and multivariate Cox regression analyses, and nomograms. Lastly, the relationship between individual hub RBPs and HCC patient overall survival (OS) was evaluated using Kaplan-Meier curves. Finally, find protein-coding genes (PCGs) related to hub RBPs were used to construct a hub RBP-PCG co-expression network.

**Results:**

In total, we identified 81 RBPs that were differentially expressed in HCC tumors relative to healthy tissues (54 upregulated, 27 downregulated). Seven prognostically-relevant hub RBPs (SMG5, BOP1, LIN28B, RNF17, ANG, LARP1B, and NR0B1) were then used to generate a prognostic model, after which HCC patients were separated into high- and low-risk groups based upon resultant risk score values. In both the training and test datasets, we found that high-risk HCC patients exhibited decreased OS relative to low-risk patients, with time-dependent area under the ROC curve values of 0.801 and 0.676, respectively. This model thus exhibited good prognostic performance. We additionally generated a prognostic nomogram based upon these seven hub RBPs and found that four other genes were significantly correlated with OS.

**Conclusion:**

We herein identified a seven RBP signature that can reliably be used to predict HCC patient OS, underscoring the prognostic relevance of these genes.

## Background

Liver cancer is among the most common forms of cancer, and owing to its highly invasive nature it is the fourth leading cancer-related cause of death globally [1]. Hepatocellular carcinoma (HCC) accounts for approximately 80% of all liver cancer cases [2], and it can be difficult to reliably diagnose and treat in its early stages, as its detection is largely dependent upon imaging evaluations and biopsy. HCC treatments generally include hepatectomy, liver transplantation, radiofrequency ablation (RFA), and transcatheter arterial chemoembolization (TACE). As his disease is generally only detected when it is in an advanced stage, HCC patients generally have a poor overall prognosis [3, 4]. The identification of novel diagnostic and prognostic biomarkers associated with HCC is thus very important.

RNA binding proteins (RBPs) are a broad class of highly-conserved RNA-interacting proteins, of which roughly 60% are expressed in a tissue-specific manner [5]. Genome-wide screening analyses have detected over 1500 RBPs in the human genome. These proteins are capable of binding to diverse RNA types (including rRNAs, ncRNAs, snRNAs, miRNAs, mRNAs, tRNAs, and snoRNAs), and can serve as key post-transcriptional regulators of gene expression to maintain intracellular homeostasis [6, 7]. RBP dysregulation has been shown to be associated with oncogenesis in multiple studies [8]. For example, Lin28 is an oncogenic RBP that has been found to promote the metastatic progression of diverse human cancers [9]. The RBP Musashi1 (Msi1) has been shown to promote glioma progression when its normal interactions with miR-137 are disrupted [10]. PUM2 is an RBP that is overexpressed in breast cancer and to be negatively correlated with OS and a lack of tumor recurrence in these patients [11]. The RBP insulin-like growth factor 2 mRNA-binding protein 3 (IGF2BP3) has similarly been found to be overexpressed in mixed-lineage leukemia–rearranged (MLL rearranged) B-acute lymphoblastic leukemia (B-ALL) and to be associated with poorer outcomes and higher recurrence risks in these patients [12]. There is also specific evidence linking certain RBPs to liver cancer. For example, Sorbin and SH3 domain containing 2 (RBPSORBS2) expression is reduced in HCC patients and associated with a poor prognosis. This RBP is believed to function via regulating RORA expression to control liver cancer onset and metastasis [13]. RBM3 is an RBP capable of promoting HCC cell proliferation owing to its ability to regulate SCD-CircRNA2 production, with RBM3 overexpression being linked to reduced OS and decreased recurrence-free survival (RFS) in HCC patients [14]. While these findings are informative, few studies to date have systematically evaluated RBP expression patterns in liver cancer.

In the present study, we downloaded HCC patient gene expression and clinical data from The Cancer Genome Atlas (TCGA) database, after which we used these data to identify RBPs that were differentially expressed in HCC tumor tissues relative to healthy normal tissues. We further explored the functional roles of these RBPs through protein-protein interaction (PPI) network, gene ontology (GO) enrichment analyses, and Kyoto gene and genome encyclopedia (KEGG) pathway analyses. We also constructed a prognostic model based upon seven key hub RBPs, identifying them as potentially viable diagnostic and prognostic biomarkers of HCC.

## Methods

### Data collection

We downloaded level 3 mRNA expression and clinical data from 374 HCC and 50 normal control samples from the TCGA – liver HCC dataset (TCGA-LIHC)(https://portal.gdc.cancer.gov/).

### Differentially expressed RBP identification

Appropriate R packages were used to standardize data by excluding genes with an average count < 1. Differentially expressed RBPs were then identified using R (v3.6.0) with the following criteria: | log2FC | ≥ 1 and FDR < 0.05.

### Functional enrichment analyses

In order to explore the functional roles of these differentially expressed RBPs, they were next separated into those that were upregulated and downregulated in HCC. The clusterProfiler R package [15] was then used to conduct GO and KEGG pathway enrichment [16] analyses on these two groups of RBPs, with *P* < 0.05 and FDR < 0.05 being used as significance thresholds.

### PPI network construction and analysis

The STRING database (http://www.string-db.org/) [17] was used to assess interactions between proteins related to these differentially expressed RBPs, with Cytoscape v3.7.1 being used to construct a PPI network. The MCODE plugin was then used to identify key modules and hub genes within this network based on the following criteria: degree cutoff = 5, node score cutoff = 0.2, k-core = 5, max.depth = 100 truncation standard, and *P* < 0.05 was the significance threshold.

### Evaluation of hub gene prognostic relevance

Follow-up analyses incorporated all HCC patients surviving for at least 30 days. Hub RBPs associated with patient prognosis were identified through univariate Cox regression analyses, with patients being randomly separated into training and test cohorts. RBPs identified in these initial analyses were then assessed via a multivariate stepwise Cox regression approach to identify hub RBPs individually associated with HCC patient OS.

### Prognostic risk score model construction and analysis

A prognostic risk score model was constructed using patients in the training cohort (*n* = 172) based upon multivariate stepwise Cox regression model coefficient (β) values for selected hub RBPs. Risk scores for *n* hub genes were computed as follows: risk score = (β-mRNA1 * expression mRNA1) + (β-mRNA2 * expression mRNA2) + (β-mRNA3 * expressionmRNA3) + (β-mRNA*n* * expression mRNA*n*). The R *survival* and *Survminer* packages were used to select the optimal risk score cutoff values [18]. HCC patients were then separated into low- and high-risk groups based upon median risk score values. The OS of patients in these two risk groups was then compared using Kaplan-Meier survival curves and log-rank sum tests with the R *survival* package. The *Survival ROC* package was additionally utilized for time-related ROC analyses assessing the value of individual hub RBPs as predictors of patient OS. These analyses were then repeated in the test group of patients.

### Nomogram construction

Nomograms have been used to predict outcomes in patients with a range of cancer types [19]. In order to construct a nomogram in the present study, the multivariate Cox analysis results pertaining to hub RBPs were used to construct line diagrams. Total nomogram scores were then used to predict 1-, 3-, and 5-year OS in HCC patients in both the training and test cohorts.

### Assessment of the correlation between risk scores and clinical characteristics

Logistic regression analyses of the entire TCGA-LIHC cohort were used to analyze the relationship between risk scores and HCC clinical characteristics. These clinical parameters included age, fender, AFP, Hepatitis B or C status, and alcohol consumption. *P* < 0.05 was the significance threshold.

### Assessment of the independent prognostic relevance of risk scores

The independent prognostic relevance of hub RBP risk scores, age, sex, tumor grade, tumor stage, and TNM stage was analyzed through univariate and multivariate Cox regression analysis. TCGA entries with incomplete data were omitted from these analyses. P < 0.05 was the significance threshold.

### Prognostic RBP validation

To analyze the prognostic relevance of identified hub RBPs in HCC patients, we utilized Kaplan-Meier curves. The *survival* R package was used to compute *P*-values corresponding to these curves via the log-rank test, with P < 0.05 as the significance threshold.

### Hub RBP-PCG co-expression network construction

A co-expression network of hub RBPs and protein-coding genes (PCGs) was additionally constructed in order to further explore the potential mechanisms whereby hub RBPs influence tumor development. Pearson correlation coefficients between RBP and PCG expression levels were calculated, and when these coefficients were > 0.5 or < − 0.5 with a *p*-value < 0.01, this was indicative of a significant correlation. An RBP-PCG co-expression network was constructed using these values, and GO and KEGG enrichment analyses of PCGs were performed.

## Results

### Differentially expressed RBP identification

**Fig. 1:**
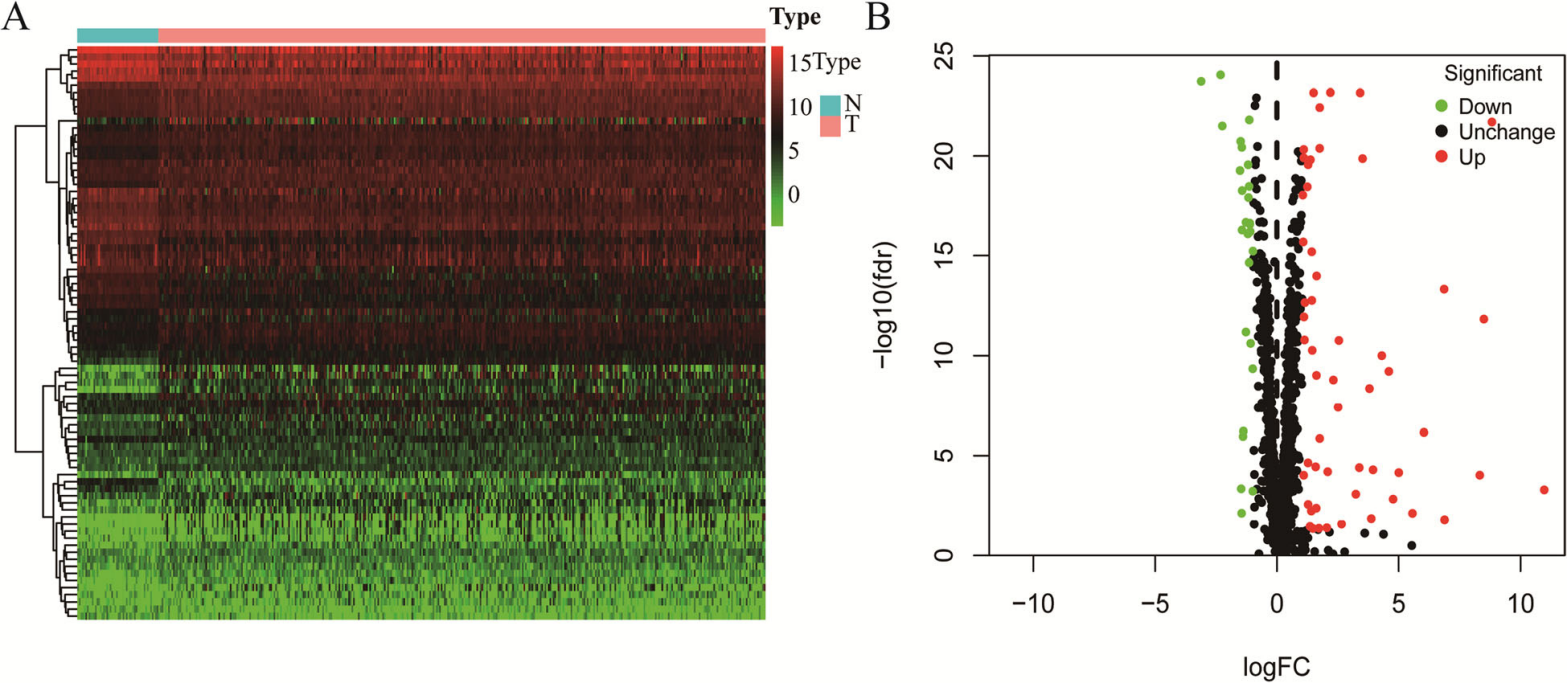
Volcano plots and Heat maps of differentially expressed RBPs. **a** Heat map; **b** Volcano plot

In total, we evaluated the expression of 1542 different RBPs in 374 HCC tumors and 50 normal tissue samples [6]. Of these, we identified 81 differentially expressed RBPs, including 54 and 27 that were upregulated and downregulated, respectively (|log2FC| > 1.0 and *P* < 0.05) (Fig. 1).

### Functional enrichment analyses

**Fig. 2:**
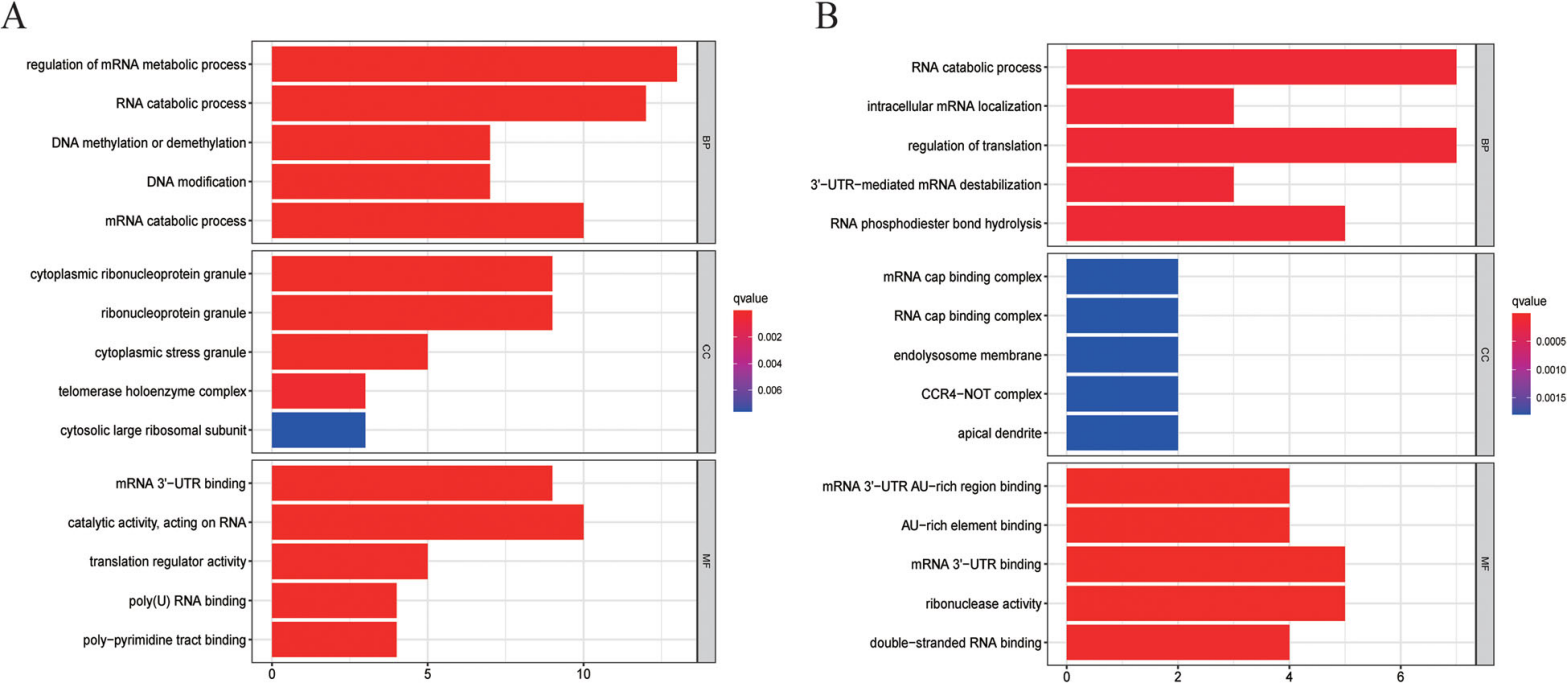
The top 5 significantly enriched GO annotations associated with differentially expressed RBPs. **a** Up-regulated RBPs; **b** down-regulated RBPs. Where CC stands for cellular component, BP for the biological process, and MF for molecular function

GO and KEGG analyses were next used to assess the potential functional roles of up- and down-regulated RBPs in HCC patients. GO analyses revealed upregulated RBPs to be enriched for roles in mRNA metabolic processes, RNA catabolic processes, DNA methylation or demethylation, DNA modification, and mRNA catabolic processes (Fig. 2a). In contrast, downregulated RBPs were enriched for roles in RNA catabolic processes, intracellular mRNA localization, translational regulation, 3′ − UTR − mediated mRNA destabilization, and RNA phosphodiester bond hydrolysis (Fig. 2b). With respect to molecular functions, upregulated RBPs were enriched in mRNA 3′ − UTR binding, catalytic activity, acting on RNA, translation regulator activity, poly(U) RNA binding, and poly−pyrimidine tract binding (Fig. 2a), whereas downregulated RBPs were enriched in mRNA 3′ − UTR AU − rich region binding, AU − rich element binding, mRNA 3′ − UTR binding, ribonuclease activity and double−stranded RNA binding (Fig. 2b). Upregulated RBPs were additionally enriched in the cytoplasmic ribonucleoprotein granule, ribonucleoprotein granule, cytoplasmic stress granule, telomerase holoenzyme complex, and cytosolic large ribosomal subunit compartments (Fig. 2a), while downregulated RBPs were primarily enriched in mRNA cap-binding complex, RNA cap-binding complex, endolysosome membrane, and apical dendrite compartments (Fig. 2b). Upregulated RBPs were additionally enriched in the mRNA surveillance pathway, microRNAs in cancer, RNA transport, RNA degradation, DNA replication, and cysteine and methionine metabolism KEGG pathways (Table 1), whereas downregulated RBPs were enriched in the influenza A, mRNA surveillance, and Hepatitis C pathways (Table 1).

**Table 1:** Analysis of KEGG pathway of aberrantly expressed RBPs

Term	Count	*p*-value
Up-regulated RBPs		
mRNA surveillance pathway	5	1.71E-06
MicroRNAs in cancer	5	0.00061399
RNA transport	4	0.00072374
RNA degradation	3	0.000789978
DNA replication	2	0.00317763
Cysteine and methionine metabolism	2	0.005824133
Down-regulated RBPs		
Influenza A	4	0.000219498
mRNA surveillance pathway	3	0.000578381
Hepatitis C	3	0.002696851

### PPI network construction and analysis

**Fig. 3:**
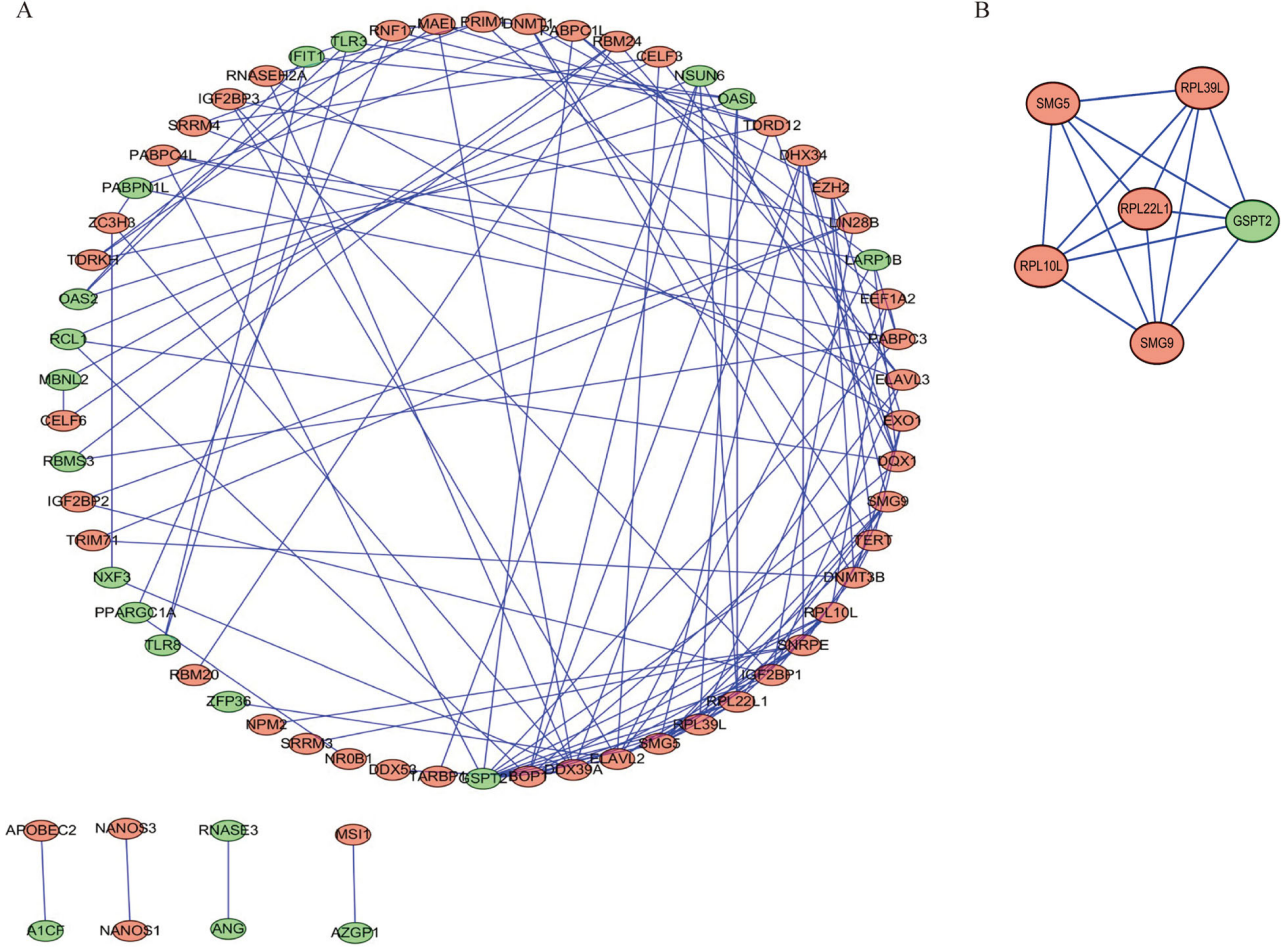
Analysis of modules and network of protein-protein interaction. **a** The network of protein-protein interaction of differentially expressed RBPs; **b** A critical module from the network of PPI. Red circles: > 2-fold up-regulation Green circles: > 2-fold down-regulation

We next utilized Cytoscape (3.7.1) to construct a PPI network based on the STRING database. The resultant network incorporated 66 nodes and 127 edges (Fig. 3a). Key co-expressed modules within this network were then identified using the MCODE plugin (Fig. 3b). Functional enrichment analyses revealed that hub RBPs within this network were enriched in mRNA catabolic processes, RNA catabolic processes, mRNA surveillance pathways, and ribosome pathways.

### Identification of hub RBPs associated with HCC patient prognosis

**Fig. 4:**
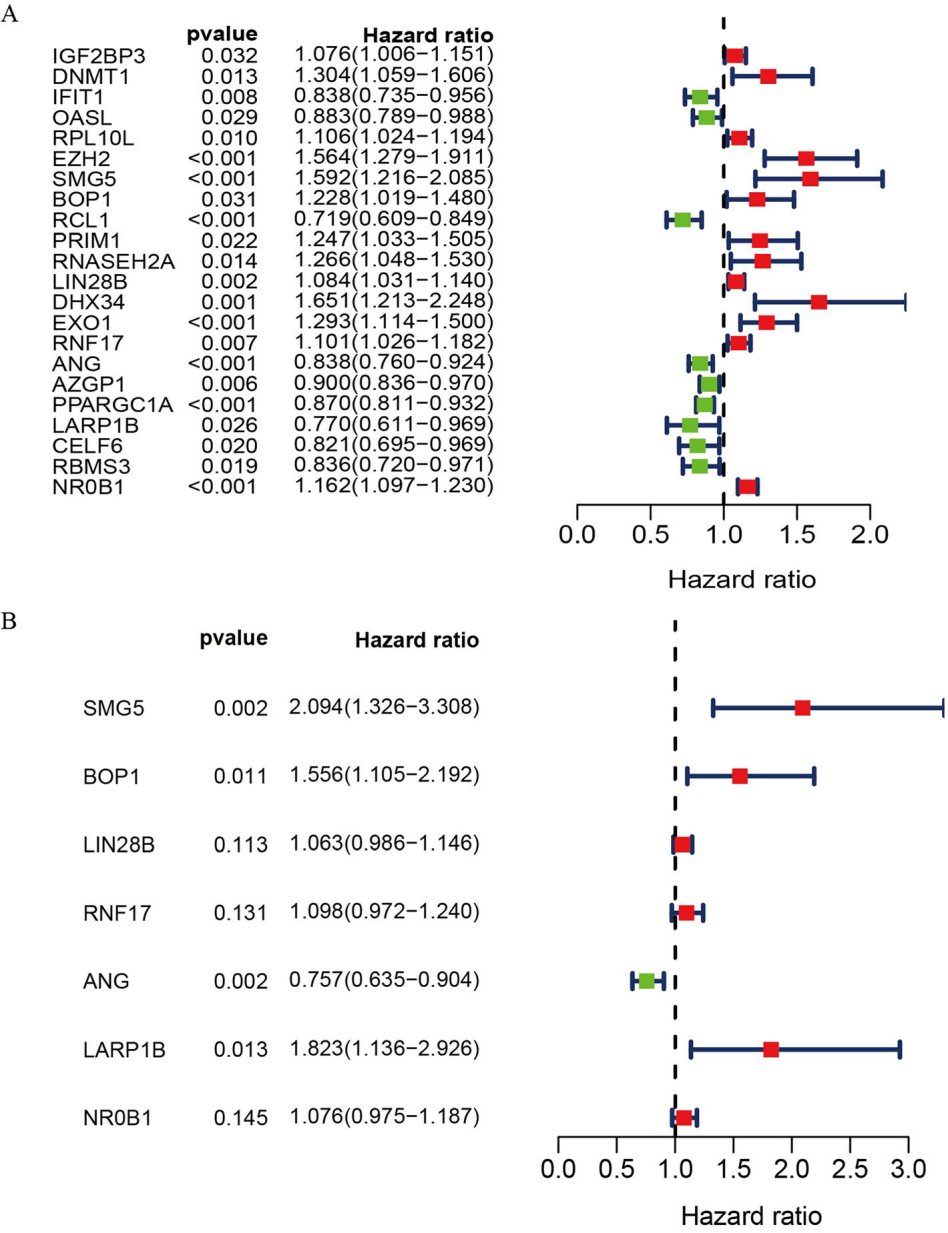
Forest plot for univariate and multivariate Cox regression analyses of HCC patients. **a** Univariate Cox regression analysis for the hub RBPs identification in the TCGA patient cohort; **b** Multivariate Cox regression analysis for the identification of hub RBPs related to patient prognosis in the training set (*n* = 172)

We next randomly separated 343 total HCC patients in the TCGA-LIHC dataset that had survived for a minimum of 30 days into a training cohort (*n* = 172) and a test cohort (*n* = 171). These two patient cohorts were then used to conduct survival analyses, leading us to identify 22 hub RBPs that were associated with patient OS (Fig. 4a). A further multivariate Cox regression analysis determined that seven of these hub RBPs (SMG5, BOP1, LIN28B, RNF17, ANG, LARP1B, NR0B1) were independently associated with HCC patient OS (Fig. 4b).

### Construction and validation of a hub RBP-based prognostic model

**Fig. 5:**
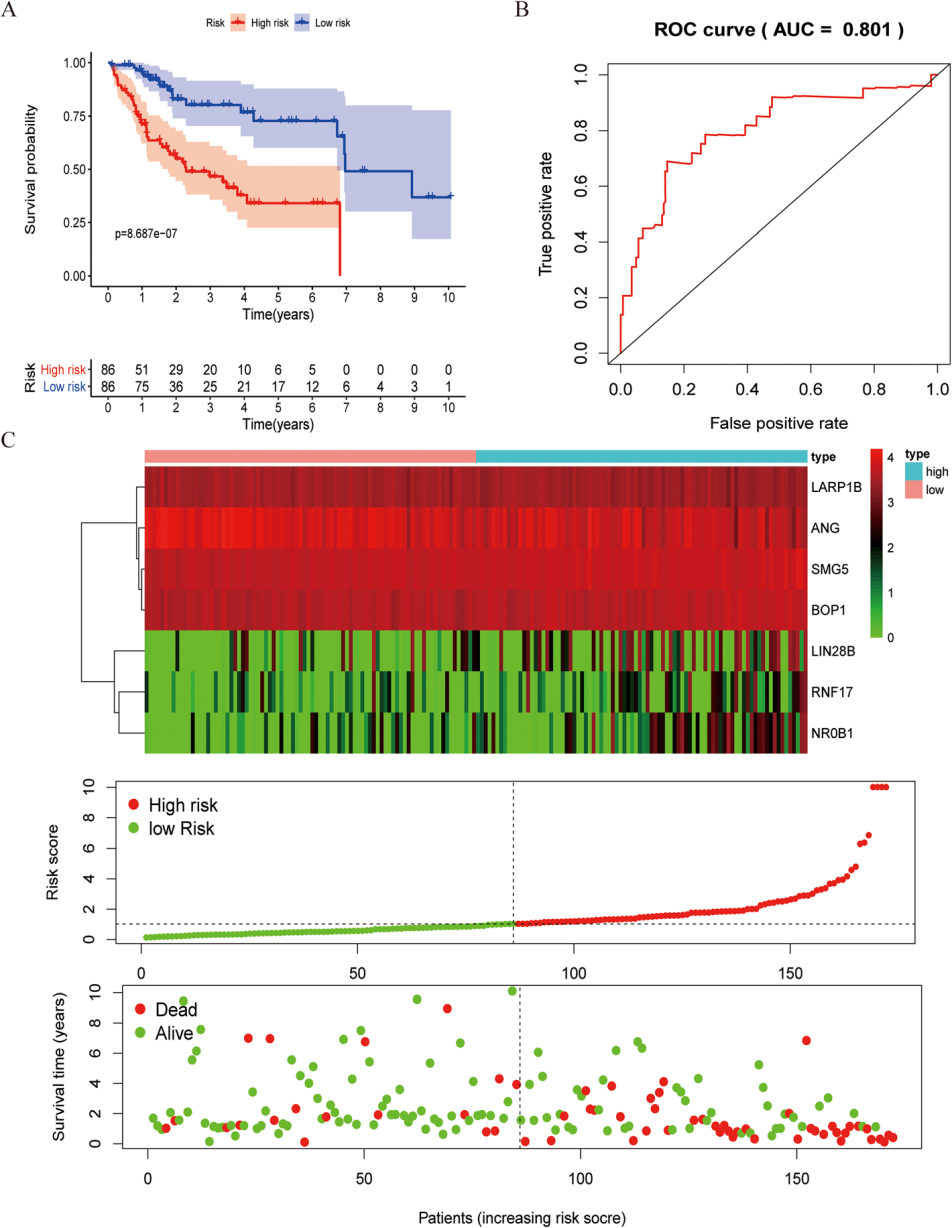
Risk score analysis of a seven hub RBP-based prognostic model in the training set (*n* = 172). **a** Survival curves for high- and low-risk patient groups; **b** ROC curves used to predict OS on the basis of risk score; **c** Expression survival status, distribution of risk score, and heat map

**Fig. 6:**
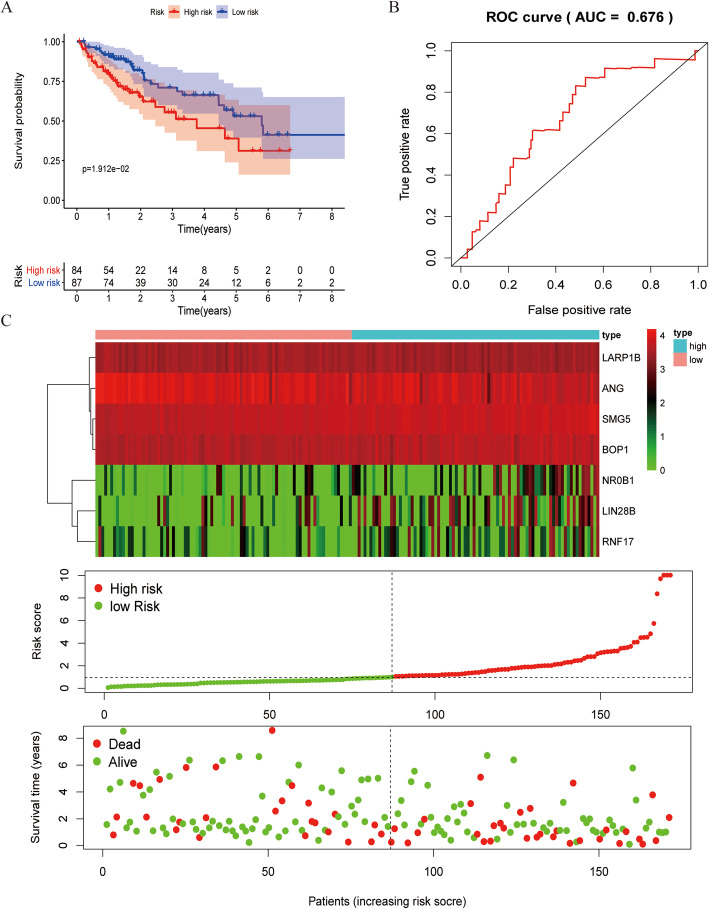
Analysis of risk score of a seven hub RBP-based prognostic model in the testing set (*n* = 171). **a** Survival curves for high- and low-risk patient groups; **b** ROC curves used to predict OS on the basis of risk score; **c** Expression survival status, distribution of risk score, and heat map

We next utilized these seven independent prognostic hub RBPs to construct a prognostic risk score model as follows: risk score = 0.7291*ExpressionSMG5 + 0.4424*ExpressionBOP1 + 0.0610*ExpressionLIN28B + 0.0936*ExpressionRNF17 + (0.2779)*ExpressionANG+ 0.6005*ExpressionLARP1B + 0.0731*ExpressionNR0B1. Risk scores for each patient in the training set were then calculated, and the *Survminer* R package was used to calculate the median risk score in this patient cohort. This median value was used to stratify patients into low- and high-risk groups, and survival outcomes between these groups were then compared via Kaplan-Meier survival and time-dependent ROC analyses. This analysis confirmed that the OS of HCC patients in the high-risk group was significantly reduced relative to that of patients in the low-risk group (Fig. 5a), with an area under the ROC curve value of 0.801 for this seven RBP risk score model (Fig. 5b), consistent with its moderate diagnostic performance. In Fig. 5c, mRNA expression levels, survival status, and risk score values for patients in the low- and high-risk groups are shown. We then utilized this same risk score formula to analyze patients in the test cohort (*n* = 171) (Fig. 6a-c). Consistent with the above results, HCC patients in the low-risk group exhibited an OS that was significantly longer than that of patients in the high-risk group, with an area under the ROC curve of 0.676. This thus indicates that our prognostic model was able to successfully predict HCC patient survival outcomes.

### Construction of a hub RBP-based prognostic nomogram

**Fig. 7:**
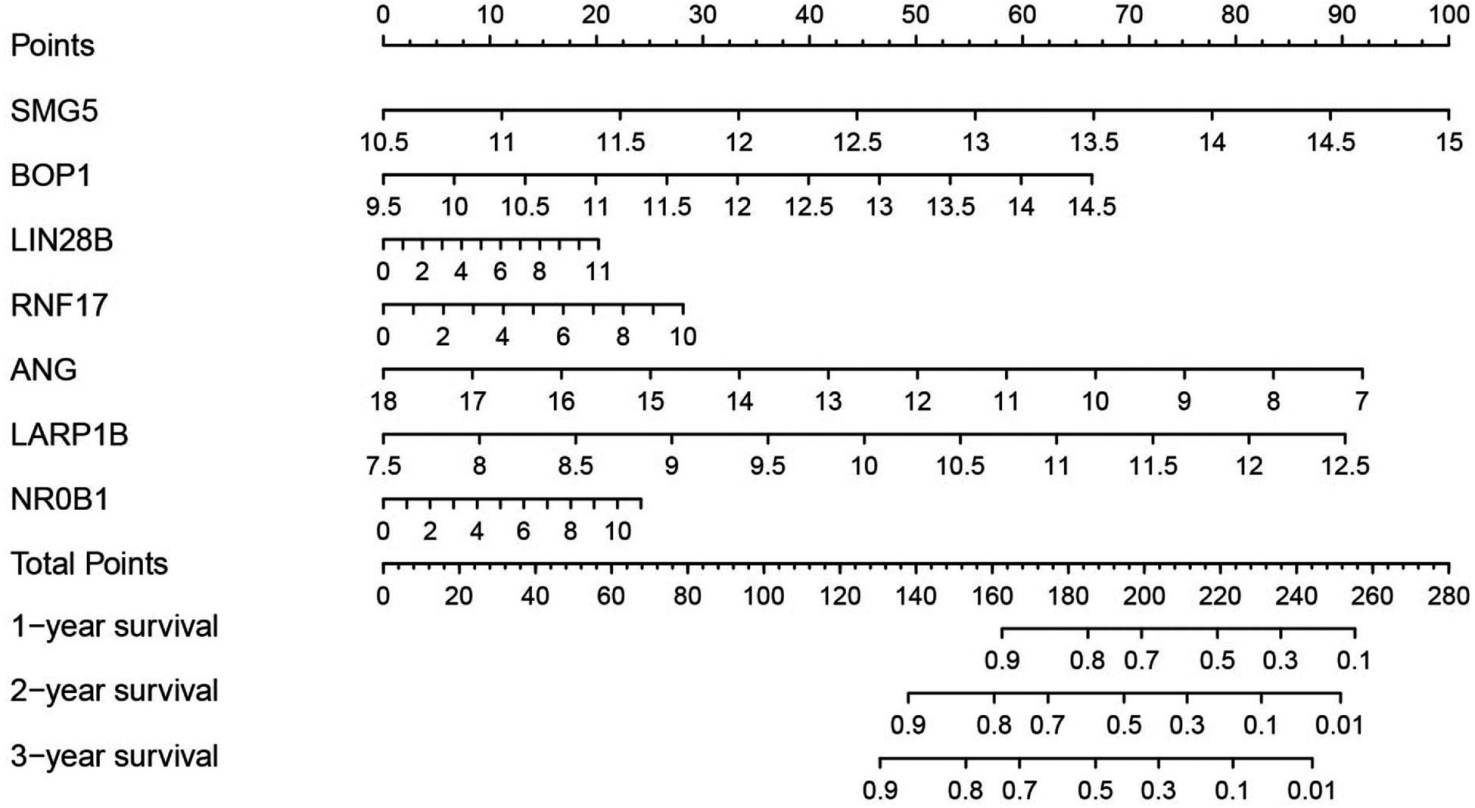
Nomogram for the prediction of OS in LIHC patients at 1, 3, and 5 years in the training set (*n* = 172)

A nomogram incorporating the results of the above multivariate Cox regression analysis pertaining to the seven hub RBPs was next constructed and used to predict 1-, 3-, and 5-year HCC patient OS (Fig. 7) in our training dataset. This analysis revealed that patient 1-, 3-, and 5-year OS declined as risk scores increased, consistent with our above results, confirming the prognostic value of this risk nomogram.

### The relationship between risk scores and clinical parameters

Logistic regression analyses were used to assess the relationship between risk scores and HCC clinical characteristics, revealing that high risk scores were associated with low histological grade (G3–4 vs G1–2, OR = 2.060) and high AFP levels (> 20 ng/mL vs < =20 ng/mL, OR = 1.986) (*P* < 0.05). In contrast, these scores were unrelated to hepatitis status, vascular invasion, or alcohol intake (Table 2).

**Table 2:** The relationship between risk scores and HCC clinical characteristics

parameter	count (N)	Odds ratio in riskScore	*p*-Value
age(>60vs < =60)	343	1.279 (0.837–1.958)	0.256
Sex (male vs female)	343	1.164 (0.740–1.836)	0.511
AFP(> 20 ng/mLvs<=20 ng/mL)	260	1.986 (1.215–3.270)	**0.006**
Hepatitis B or C (Yes vs No)	315	0.670 (0.428–1.046)	0.079
Alcohol consumption (Yes vs No)	315	1.161 (0.731–1.846)	0.528
Cirrhosis status (Yes vs No)	199	0.612 (0.334–1.110)	0.108
Vascular invasion (Yes vs No)	289	1.042 (0.642–1.692)	0.868
Grade (G3–4 vs G1–2)	338	2.060 (1.316–3.249)	**0.002**
Stage (Stage III-IV vs Stage I-II)	321	1.649 (0.997–2.751)	0.053
T (T3–4 vs T1–2)	340	1.541 (0.946–2.527)	0.084
N (N1 vs N0)	242	2.017 (0.191–43.740)	0.569
M (M1 vs M0)	248	2.016 (0.191–43.723)	0.569

Bold values indicate *P* < 0.05

### RBP risk scores independently predict HCC patient prognosis

**Fig. 8:**
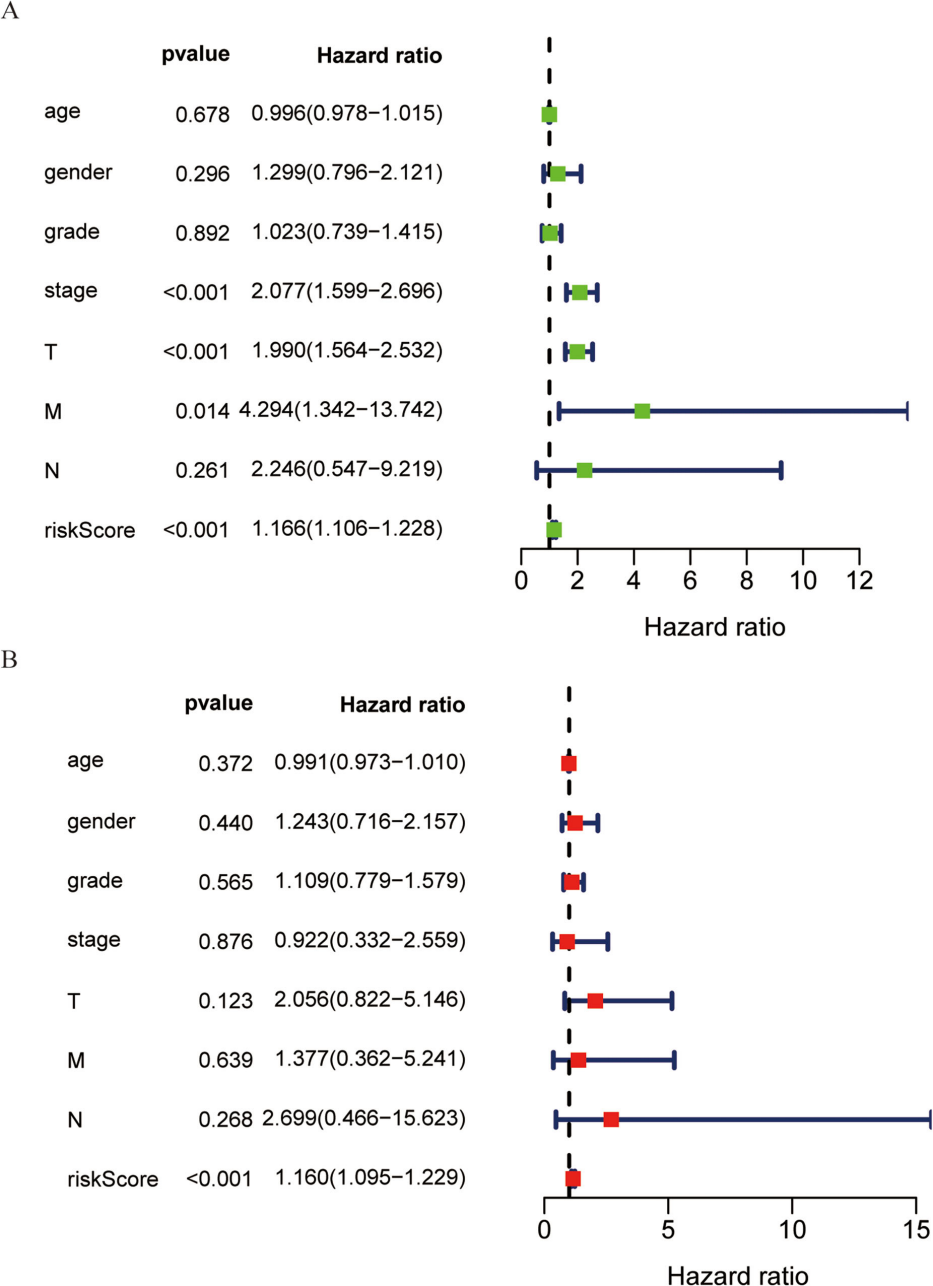
Univariate and multivariate analyses of the correlation between risk score and OS. **a** Univariate Cox analyses; **b** multivariate Cox analysis

We next conducted univariate Cox analyses or factors associated with prognosis in 226 patients that survived for a minimum of 30 days and for whom complete clinical data were available. These analyses revealed that cancer tissue stage, T stage, and risk scores were all associated with HCC patient OS (*P* < 0.001) (Fig. 8a). Subsequent multivariate Cox analysis confirmed that the RBP risk score was an independent predictor of HCC patient OS, with a hazard ratio (HR) of 1.160 and a 95% confidence interval of 1.095–1.229 (*P* = 4.305E-07) (Fig. 8b).

### Validation of hub RBP prognostic value

**Fig. 9:**
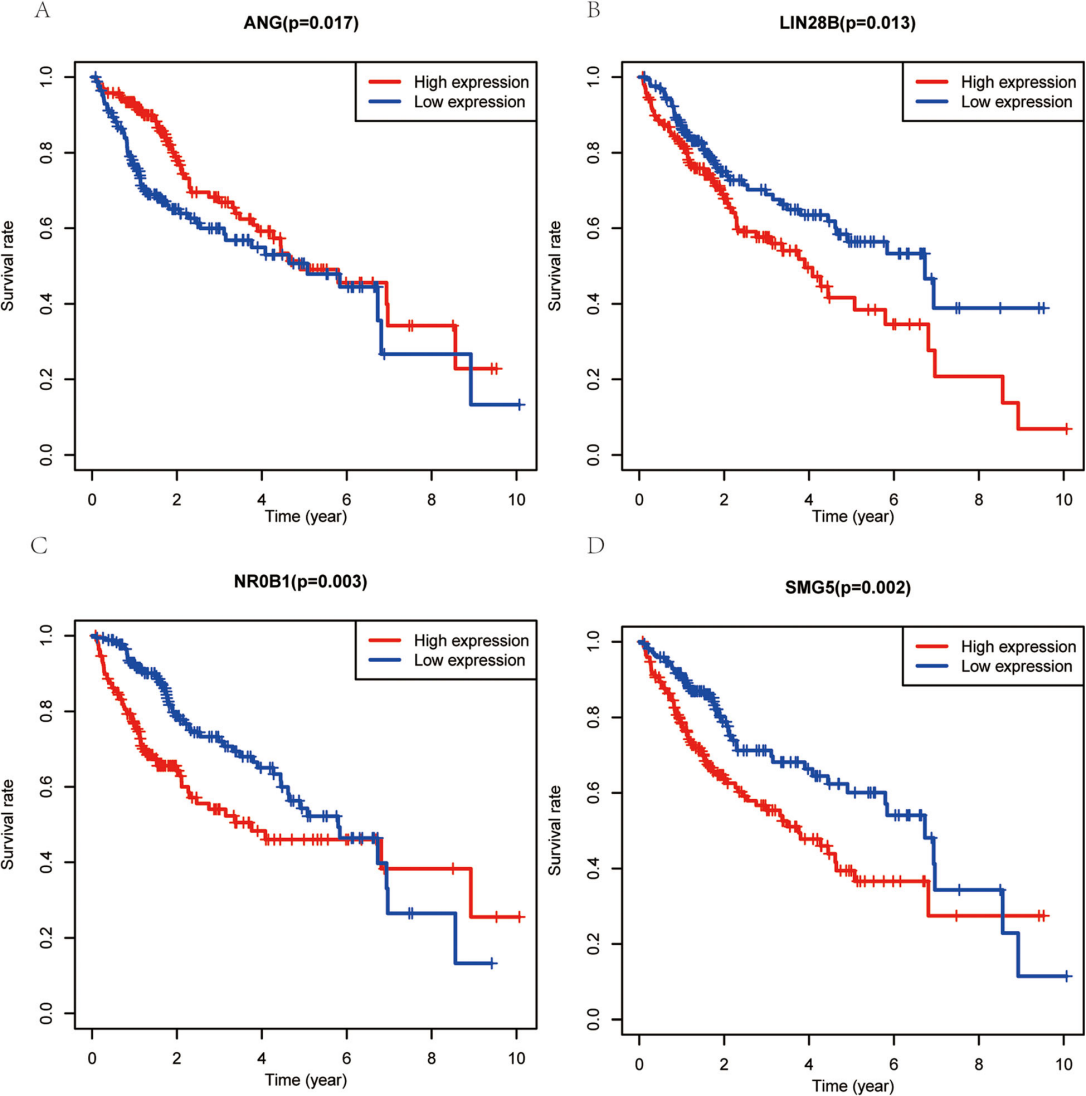
Validation of the hub RBPs prognostic value in HCC patients in the TCGA cohort

Lastly, the relationship between identified hub RBPs and HCC patient OS was evaluated using the Kaplan-Meier plotter database. This analysis confirmed that 4/7 hub RBPs (ANG, LIN28B, SMG5, and NR0B1) were significantly associated with HCC patient OS, with respective *P*-values of 0.017, 0.013, 0.002, and 0.003 (Fig. 9a-d).

### SMG5-PCG co-expression network analysis

A correlation analysis of SMG5 and PCGs revealed that there were 3756 total PCGs correlated with SMG5, of which 7 were negatively correlated and 3749 were positively correlated. The top five PCGs positively correlated with SMG5 expression were ISG20L2, DENND4B, UBQLN4, PI4KB, and SLC39A1 (Table 3), while the top five PCGs negatively correlated with SMG5 expression were TTC36, CLEC4M, FCN2, MFSD2, and MT1X (Table 3). GO and KEGG analyses of these SMG5-related PCGs revealed them to be primarily enriched in the mTOR, AMPK, VEGF, and hepatitis B signaling pathways, indicating that they are closely related to tumor development.

**Table 3:** The top 10 PCGs correlated with SMG5 expression

Correlated PCG	Spearman’s Correlation	*p*-value
ISG20L2	0.8	9.48E-96
DENND4B	0.8	8.02E-96
UBQLN4	0.801	4.57E-96
PI4KB	0.805	1.19E-97
SLC39A1	0.808	5.05E-99
TTC36	−0.563	7.86E-37
CLEC4M	−0.534	1.07E-32
FCN2	−0.514	5.75E-30
MFSD2A	−0.511	1.53E-29
MT1X	−0.508	3.48E-29

## Discussion

While available treatments for HCC have improved significantly in recent years [20], it remains a condition associated with high rates of morbidity and mortality [21]. As such, it is essential that novel diagnostic and prognostic biomarkers of HCC be identified in order to improve patient outcomes.

RBP dysregulation has been shown to be a hallmark of many tumor types [8]. In gliomas [10], breast cancer [11], and B-ALL [12], RBPs have been found to be directly related to tumor development and patient prognosis. In the present study, we identified 81 RBPs that were differentially expressed in HCC tissues relative to healthy control tissues in the TCGA-LIHC dataset. We analyzed the biological roles of these RBPs through functional enrichment analyses and by constructing a PPI network, after which we employed Cox regression analyses, survival analyses, and time-dependent ROC analyses of key hub RBPs within this network to construct a prognostic risk model. This model was capable of predicting HCC patient OS based upon the intratumoral expression of seven key RBPs. As such, our results highlight these RBPs as novel prognostic biomarkers of HCC, and additionally identify these genes as potential diagnostic or therapeutic targets. These differentially expressed RBPs were found to be functionally enriched in pathways relating to the regulation of mRNA metabolism, RNA catabolism, DNA methylation or demethylation, DNA modification, translation regulation, mRNA3’-UTR binding, ribonuclease activity,, ribonucleoprotein granule, telomerase holoenzyme complex, and dsRNA binding. It has been reported that human ribosomal protein S3 (RPS3) regulates the expression of silent information regulator 1 (SIRT1) after transcription to promote liver cancer [22]. IGF2 mRNA-binding proteins (IGF2BPs) can specifically bind to the lncRNA HULC (Highly Up-regulated in Liver Cancer) HULC, thereby controlling its expression [23]. Polypyrimidine tract-binding protein 1 (PTBP1) is highly expressed in hepatocellular carcinoma and promotes the translation of cyclin D3 (CCND3) via interacting with the 5′-untranslated region (5′-UTR) of its mRNA, thereby playing a role in the development of hepatocellular carcinoma [24].RBPs are capable of specifically binding to conserved 3′-UTR sequences in target mRNAs, thereby modulating their stability and subsequent translation [25, 26]. Appropriate regulation of DNA modification is essential to ensure that chromosomes replicate correctly, and that genes are expressed or silenced in a context-appropriate manner [27]. Promoter or gene body hypermethylation can lead to the inactivation of key tumor suppressor genes, and methylation-based epigenetic silencing of specific genes is a hallmark of many forms of cancer [28]. There are also many studies that show that telomerase plays an important role in the development of liver cirrhosis and liver cancer [29]. Our KEGG pathway analyses further suggested that these dysregulated RBPs may be linked to HCC onset and progression owing to their ability to influence mRNA monitoring pathway, microRNA, RNA transport, RNA degradation, and DNA replication pathway. For example, microRNAs have been shown to play an important role in post-transcriptional regulation of gene expression. Indeed, microRNA dysregulation is thought to be associated with tumor suppressor gene inactivation and oncogene activation in liver cancer [30]. The mRNA monitoring pathway is essential for maintaining homeostasis such that when this regulation is disrupted it can facilitate tumor pathogenesis [31]. As such, these mechansims may explain how differentially expressed RBPs are associated with the development of liver cancer.

Through Cox regression analyses, we detected seven key RBPs that were associated with HCC patient prognosis, including SMG5, BOP1, LIN28B, RNF17, ANG, and LARP1B. These seven hub RBPs exhibit telomerase RNA binding, ribonucleoprotein complex binding, DNA binding, ribonuclease, DNA-binding transcription factor, and RNA polymerase II-specific functions [32–34]. They are additionally involved in the regulation of telomere maintenance, the cell cycle, RNA 3′-end processing, cell migration, and in the negative regulation of transcription [33, 35–38]. These genes are closely linked to tumor development. In prior studies, BOP1 has been shown to promote liver cancer development via driving epithelial to mesenchymal transition [39]. Lin28b is a miR-125a target gene that, when downregulated, can inhibit liver cancer cell proliferation [40], NR0B1 (also called DAX-1) can inhibit the proliferation of liver cancer cells by suppressing the transcriptional activity of β-catenin [41]. In HCC patients, plasma samples contain high levels of angiopoietin-1 (Ang-1), and patients with low angiopoietin-2 (Ang-2) levels exhibit better OS [42]. We then employed a multivariate stepwise Cox regression analysis to establish a risk model incorporating these seven hub RBPs that can be used to predict HCC patient prognosis. Time-dependent ROC curve analyses revealed that these seven genes offered good diagnostic ability, and that our risk model could be readily used to identify HCC patients with a poor prognosis. However, few studies to date have explored the molecular mechanisms whereby these hub RBPs influence HCC pathogenesis, and as such, further research is essential. We additionally constructed a nomogram capable of predicting the 1-, 3-, and 5-year HCC patient OS. In addition, we utilized Kaplan-Meier curves to assess the prognostic value of these seven hub RBPs, with four of them being found to be associated with patient outcomes. We also constructed a co-expression network of SMG5 and correlated PCGs in order to discover its potential downstream target genes and to explore the possible regulation of RBPs involved in the development of liver cancer. We found that PCGs associated with SMG5 were related to tumorigenesis. Closely-related genes such as UBQLN4 are upregulated in aggressive tumors and promote non-homologous end binding (NHEJ) during DSB repair, resulting in DNA mismatches [43]. HCC patients exhibiting CLEC4M overexpression have a better OS, and CLEC4M overexpression inhibits the proliferation of liver cancer cells and promotes their apoptotic death [44]. KEGG pathway enrichment analyses revealed that these PCGs were enriched in the mTOR, AMPK, and VEGF signaling pathways, all of which are closely linked to cancer development and progression [45–47]. These pathways may thus be one mechanism whereby these RBPs participate in the occurrence and development of liver cancer and other malignancies. As such, these differentially expressed hub RBPs offer clear value in the assessment of HCC patients and may represent viable therapeutic targets. Despite the lack of effective adjuvant therapy for liver cancer, the application of targeted drugs provides a promising opportunity for imporiving the prognosis of those affected by this disease [48].

## Conclusions

In summary, in the present study we developed a predictive model of HCC patient survival based upon the expression of seven key RBPs within tumor tissues. While this model exhibited significant prognostic value, this study is limited by the fact that it is solely based upon data within the TCGA database and lacks any external validation. In addition, we have not explored the functional roles of these RBPs in the context of HCC, and as such, future in vitro and in vivo analyses will be necessary to confirm and expand upon our findings. In addition, candidate RBPs may provide insight into the regulation of HCC while offering value as prognostic biomarkers.

## Data Availability

The datasets analysed during the current study are available in the The Cancer Genome Atlas database (https://portal.gdc.cancer.gov/).

## Abbreviations

HCC: hepatocellular carcinoma
RBPs: RNA-binding proteins
TCGA-LIHC: the Cancer Genome Atlas - liver HCC
OS: overall survival
PCGs: protein-coding genes
RFA: recurrence-free survival
TACE: transcatheter arterial chemoembolization
RFS: recurrence-free survival
TCGA: The Cancer Genome Atlas
PPI: protein-protein interaction
GO: gene ontology
KEGG: Kyoto gene and genome encyclopedia
